# Leishmanicidal and fungicidal activity of lipases obtained from endophytic fungi extracts

**DOI:** 10.1371/journal.pone.0196796

**Published:** 2018-06-18

**Authors:** Daniela Ribeiro Alves, Selene Maia de Morais, Fernanda Tomiotto-Pellissier, Fábio Roger Vasconcelos, Francisco das Chagas Oliveira Freire, Isaac Neto Goes da Silva, Alan Henrique Depieri Cataneo, Milena Menegazzo Miranda-Sapla, Gustavo Adolfo Saavedra Pinto, Ivete Conchon-Costa, Arlindo de Alencar Araripe Noronha, Wander Rogério Pavanelli

**Affiliations:** 1 Veterinarian Sciences Post Graduation Program, State University of Ceará, Campus Itaperi, Fortaleza, Ceará, Brazil; 2 Empresa Brasileira de Pesquisa Agropecuária (EMBRAPA)–Agroindústria Tropical, Planalto do Pici, Fortaleza, Ceará, Brazil; 3 Pathological Sciences, State University of Londrina, Campus Universitário, Londrina, Paraná, Brazil; 4 Animal Physiology Laboratory, Department of Animal Science, Federal University of Ceará, Campus Pici, Fortaleza, Brazil; Tallinn University of Technology, ESTONIA

## Abstract

This work describes the production of lipases from endophytic fungi: *Vermisporium-like*, *Emericella nidulans*, *Dichotomophtora portulacae* and *D*. *boerhaaviae* and the biological activity against the dermatophyte fungi *Malassezia* sp and *Microsporum canis* and the parasite *Leishmania amazonensis*. All fungal enzymes extract showed lipolysis action in the media that contains long carbon chain lipids. The proteomic analysis of lipases exhibits several molecules mostly ranging in size from 220 to 20 kDa, with clear differences in protein profile’s yield. All fungal enzymes were competent to eliminate promastigote forms of *Leishmania amazonensis* at 5 mg.mL^-1^. The antileishmanial activity of lipases from *Vermisporium-like*, *E*. *nidulans*, *D*. *portulacae* and *D*. *boerhaaviae* in amastigote forms, promoted the reduction in viability of 78.88, 39.65, 63.17 and 98.13%, with selectivity index of 19.56, 30.68, 18.09 and 20.99. In relation to antifungal activity, *Dichothomophtora* enzymes demonstrate best action with MFC of 14.65 μg.mL^-1^ against *Malassezia* sp and *Microsporum canis*, respectively. These results allow us to infer that lipases from entophytic fungi displays activity against dermatophyte fungi (*Malassezia* sp. and *Microsporum canis)* as well as *Leishmania*.

## Introduction

American Cutaneous Leishmaniasis is a zoonotic disease caused by protozoans of the genus *Leishmania*, and has as its main characteristic the formation of painless ulcerated lesions with intense red colored edges. The treatment of cutaneous leishmaniasis is unsatisfactory and has limited efficacy, prolonged treatment, high cost and undesirable side effects to the host, which has prompted the search for new therapeutic strategies and dogs are the main domestic reservoir of the *Leishmania* parasite [1]. Another health problem, which compromises domestic animals, are pathogens of the skin, among them *Malassezia* [2], *Microsporum canis* and *Trichophyton* [3] represent the most prevalent.

The main used drugs for cutaneous leishmaniasis treatment are pentavalent antimony salts and the most common options for treatment of those mycoses are azoderivatives, but these current chemotherapies also presents high cytotoxicity. Then, alternative therapies, more efficient and less toxic, for these diseases are needed. Therefore, natural compounds could be used alone or in combination with current antimonial or azoderivatives therapy even in cases of disease recurrence or resistance [4,5].

Fungal cell membranes are one of the most important targets for drug development [6]. The therapeutic properties of many fungi metabolites lead to the search of active compounds produced from various species. Endophytic fungi are those identified within plant systems, which were previously considered sterile environments [7]. These are essential for biological control in the production of metabolites, increased resistance of the host plant to herbivores, heat, salinized soils, drought, diseases, besides acting as genetic vectors, thus having great potential for exploitation [8]. It is believed that endophytic fungi from oleaginous plant can be a potential source of lipases.

The current biodiversity of the Northeast of Brazil has a potential to offer, through the genetic patrimony, a promising source of bioactive metabolites of pharmacological importance. In this sense, this work aims to contribute to the discovery of natural products that will present leishmanicidal and fungicidal activity, based on extracts of enzymes from endophytic fungi.

## Material and methods

### Fungi isolation

Fresh seeds of *Jatropha curcas* L. were acquired at Beckman seeds®, Fortaleza, the state of Ceará. The seeds were surface sterilized with 70% ethanol for 1 minute (min) and 1.5% sodium hypochlorite for 2 min and were then rinsed twice with sterile distilled water. Excess water was removed by using sterilized filter paper. 5 (five) seeds with or without disinfestation were added to 30 (thirty) Potato Dextrose Agar (PDA) disposable plates. After three (3) days incubated at 25 °C with a 12 h photoperiod, strains of *Vermisporium-like* and *Emericella nidulans* were isolated, identified using spore morphology and submitted to microorganism collection from Empresa Brasileira de Pesquisa Agropecuária (EMBRAPA^®^)–Agroindústria Tropical under No CMIAT232 and CMIAT233, respectively. *Dichotomophtora boerhaaviae* (CMIAT 235) was obtained from EMBRAPA endophytic fungi collection and strains of *Dichotomophtora portulacae* (CBS 149.94) was obtained from CBS^®^ Fungal Biodiversity Centre collection.

### Animal welfare

The ethics committee of Londrina State University approved the protocol for animal use (162/2016).

### Enzymatic activity plate assay—Determination of hydrolases

After 5 days of culture, pellets with 6.5 mm in diameter of each fungus was inoculated in an enzymatic media, which contained as an inductor of enzyme activity “Tween 20” (T_20_); “Tween 80” (T_80_) [9,10]; or “W”, as called by the author [11]; or “K” [12]. This modified medium (K) is composed by: 1.0 g.L^-1^ Yeast extract, 1.0 g.L^-1^ KH_2_PO_4_, 0.5 g.L^-1^MgSO_4_.7H_2_O, 1.0 g.L^-1^ (NH_4_)_2_SO_4_ and 20.0 g.L^-1^ bacteriological agar.

All the media was sterilized at 121°C for 15 minutes and the medium “K” was cooled to 60 °C and then the following compounds were added: 10.0 g.L^-1^ of egg lecithin, CaCI_2_ 100.0 mM, and 2.0 mL of aqueous solution 0.1% Rhodamine B (previously sterilized by filtration through 0.22 μm syringe filter). Each media was emulsified with intense shaking by 10 minutes and then 20.0 mL distributed in each 90 mm diameter Petri plates.

The fungi were inoculated and maintained in a bacteriological incubator for 10 days at 27 °C. The enzymatic activity (S1 Fig) of fungi was observed through the formation of precipitate crystals of calcium salts or the appearance of clear different color halos around the colonies, using stereomicroscope and microscope. Daily growths were observed and measured the size of the colony and the activity halo. The bacterial strains *Pseudomonas aeruginosa* ATCC 27853 and *Staphylococcus aureus* ATCC 25923 were used as a positive control in the detection of lipase’s presence test.

### Lipolytic activity expressed as enzymatic index (EI)

Lipase activity was observed through the presence of calcium salt crystals of the lauric acid, released by the enzyme around the formed colonies. The enzymatic index was determined as described by Hankin and Anagnostakis [13], after 03 days of subculture, using the following ratio:

EI=diameterofthecolony+thehalo/diameterofthecolony (1)

If the EI is greater than 1 the production of the enzyme of interest is better.

### Enzymes extraction assay

After 5 days of culture the fungus were selected and inoculated in 5 pellets (6.5 mm in diameter) in a 250 mL Erlenmeyer containing “W” media without agar and pH adjusted to 8.0. The flasks were then incubated at 30 °C for 7 days. It was established that *Vermisporium-like* crude extract corresponds to fungal enzyme “A”, *E*. *nidulans* crude extract to “B”, *D*. *portulaceae* crude extract to “C” and *D*. *boerhaaviae* crude extract to “D”. After the incubation period, the mycelial mass was separated by filtration through Whatman filter paper # 1 [12]. The filtrates were lyophilized then diluted to a concentration of 2.0 mg.mL^-1^ for use as enzymatic extracts in the analysis. Three bottles were inoculated for each strain.

### Total protein quantification and 1D SDS-PAGE

The concentration of soluble protein [14] was determined with Bradford reagent (Sigma-Aldrich, St Louis, MO) using bovine serum albumin (BSA) as a standard and the extracts kept at -80 °C. A protein standard (BSA) was prepared in seven dilutions. The range of the protein assay was between 0 and 2.0 mg.mL^-1^. Determination of protein concentration was performed in triplicate. Spectrophotometer Reader Nanodrop 2000 (Thermo-Fisher, Waltham, EUA) was used to measure the absorbance at 595 nm.

The crude fungal extracts were lyophilized and suspended in 2 mL of distilled water. Five volumes of ice-cold acetone were added to the crude extracts and left overnight at -20°C. Then, after centrifugation at 14000 rpm for 30 min at 4°C, the collected pellets were dialyzed, further lyophilized and suspended in sample buffer (7 M urea, 2 M thiourea, 4% 3-[(3-cholamidopropyl) dimethylammonio]-1-propanesulfonate) (CHAPS), 2% free ampholytes [IPG buffer, pH 4–7 (GE LifeSciences)], 40 mM dithiothreitol (DTT) to 25 mL distilled (H2O). Insoluble material was removed by centrifugation. Proteins extracts were evaluated according to (i) the diversity of bands; (ii) the amount of protein extracted as well as (iii) the integrity of samples. Approximately 10 μg of protein preparations were analyzed by one-dimensional electrophoresis on a denatured dodecyl-sulfate-polyacrylamide gel (12% 1D-SDS-PAGE). Staining was done by using Coomassie brilliant blue G250 (sigma) [15].

### Determination of lipolytic activity of extracts

The lipase action evaluation was based on the capacity of the extracts to induce lipolysis in the media that contain long carbon chains. The crude fungal extracts were first submitted to diffusion into K and W agar gel. After agar solidification, wells of 6.5 mm diameter were prepared and 20 μL of samples were added to each well in triplicate and incubated at 30ºC for 18 h. Lipolysis activity was identified by a light blue color halo, for the W agar and a light halo with at least one color or more for the K agar (S2 Fig).

EI=diameterofthewell+thehalo/diameterofthewell (2)

The EI was measured and compared for each sample in both media [11].

### Fungicidal assay

The minimum concentration capable of inhibiting 100% fungi growth (MIC_100_) was determined by the dilution technique (2500.0 to 2.44 μg.mL^-1^), according to CLSI method [16]. Six strains were tested (3 *Malassezia* sp. and 3 *Microsporum canis*), isolated from infected domestic animals, identified and stocked at the Microbiology section and kindly donated by VETTINGS®.

Well intervals were prepared to evaluate the sterility control of the medium; to control of fungal growth and to evaluate the positive control (TECTO^®^ RC Thiabendazole; Syngenta^®^, 4.0 mg.L^-1^).

To determinate the MIC_100_ after 5 days of incubation the plates were inspected under an inverted microscope to assure growth of the controls and minimal sterile conditions.

The Minimal fungicide concentration (MFC_100_) is that capable of killing 100% of the fungal strains and was measured by transfer 50.0 μL from wells without fungal growth and inoculates on PDA. MFC_100_ was established according to the fungus growth after incubation under the same conditions for 5 days.

### Leishmania parasite

*Leishmania (Leishmania) amazonensis* (MHOM/BR/1989/166MJO) was used in promastigote forms, in the stationary growth phase (day 5 of culture). The parasites were obtained from popliteal lymph nodes of *L*. *amazonensis*-infected BALB/c mice and maintained in 199 culture medium (GIBCO) supplemented with 10% fetal bovine serum (FBS) (GIBCO), 1M HEPES Biological Buffer (AMRESCO), 1% human urine, 1% L-glutamine (SYNTH), penicillin (10 U.mL^-1^) and streptomycin (10 μg.mL^-1^) (GIBCO) and 10% sodium bicarbonate (SYNTH). Cell cultures were incubated at 25°C in 25-cm^2^ flasks. All parasites were from a culture that was serially passed for less than 5 weeks.

### Viability of *L*. *amazonensis*-promastigote forms

The direct effect of fungal extracts against *L*. *amazonensis* was performed in 24-well microtiter plates, each well containing 1000 μl of 199 supplemented culture medium with 1 x 10^6^ promastigote forms in stationary phase with or without the fungal extracts of interest (A, B, C and D) at final concentration of 5, 2.5, 1.25 and 0.625 mg.mL^-1^. Viable promastigote concentration was determined in a CASY model TT cell counter and analyzing system (ROCHE) after 24h of treatment. In the stock solutions of fungal extracts, 1% dimethyl sulfoxide (DMSO) (GIBCO) was used as a vehicle. However, DMSO concentration did not exceed 0.5% in all experiments. Untreated parasites and vehicle only (0.5% DMSO) were included as negative controls. The plates were also inspected under an inverted microscope to assure growth of the controls and sterile conditions.

### Bone marrow-derived macrophages (BMDMs)

Bone marrow-derived macrophages were obtained as previously described by Modolell and Munder [17]. Briefly, BALB/c mice were euthanized and femur and tibia bones were dissected out. Bones were trimmed at each end, and marrow was flushed out with 3.0 mL of Dulbecco’s Modified Eagle Medium-F12 (DMEM-F12) (SIGMA) containing 100 U.mL^-1^ penicillin (GIBCO) supplemented with 10% FBS. Bone marrow was dispersed and cultured in 6-well microtiter plates at 37°C and 5% CO_2_ in DMEM-F12 supplemented with 30% of L-929 fibroblasts culture supernatant.

### BMDMs viability assay

The viability of BMDMs treated with fungal extracts was evaluated using the 3-(4,5-dimethylthiazol-2-yl)-2,5-diphenyltetrazolium bromide (MTT) assay as previously described by Mosmann [18]. BMDMs (5 × 10^5^ U.mL^-1^) were cultured in 24-well plates and preincubated with 200 μL of DMEM/F12 medium for 2 h for adherence at 37°C and 5% CO_2_. The cells were washed with PBS and then, adherent cells were incubated with different concentrations of fungal extracts A, B, C and D (5, 20 and 50 mg.mL^-1^) or with vehicle (0.5% DMSO) and maintained in culture for 24h at 37°C and 5% CO_2_. After incubation with extracts, the BMDMs culture was washed with PBS and added MTT at a final concentration of 5 μg.mL^-1^ in each well, followed by incubation for an additional 4h at 37 °C/ 5% CO_2_. The MTT formazan product was solubilized with DMSO, plates were read at 570 nm in a spectrophotometer. The results were expressed as a percentage of MTT reduction relative to the control group calculated as the following formula: (viable macrophages)% = (OD of drug-treated sample/OD of the untreated sample) x 100.

### Phagocytic assay

BMDMs (5 x 10^5^ U.mL^-1^) were cultured in 24 well plates containing 13.0 mm diameter glass coverslips and incubated with 200 μL of DMEN F-12 medium for 2 h for adherence at 37ºC/ 5% CO_2_. Adherent macrophages were infected with promastigote forms (10:1) for 2h. After infection, free promastigotes were removed by washing with PBS, and the infected cells were treated with A, B, C and D fungal extracts (5.0 mg.mL^-1^) and incubated for 24 h at 37ºC and 5% CO_2_. DMEM F-12 medium was used as a control. *L*. *amazonensis*-infected BMDMs were stained with Giemsa and 20 fields analyzed using a light microscope (magnification of 1000x, OLYMPUS, model CX31RBSFA) to determine the phagocytic index by % of infection and the number of parasites/macrophage.

### Selectivity index (SI)

The effects of fungal extracts on *L*. *amazonensis* promastigote forms and BMDMs cytotoxicity were expressed as the percentage of viable cells compared to control, and the 50% inhibitory concentration (IC_50_) was calculated by non-linear regression (GraphPad Software, v. 5.00). The degree of selectivity of fungal extracts was expressed as SI = IC_50_ of fungal extracts on BMDMs/IC_50_ of the same fungal extracts on promastigotes. Higher SI reveals greater extract’s selectivity up the parasite, rather the host. The experiments were tested in triplicate.

## Statistical analysis

Analyses were performed as triplicate data sets, from 3 independent experiments. Results were analyzed as a mean ± standard deviation. Data were analyzed by ANOVA (t-Student), followed by Tukey`s test for multiple comparisons, using the software Prism GraphPad (GraphPad Software, v. 5.00). Differences were considered statistically significant at p<0.01.

## Results and discussion

**Fig 1:**
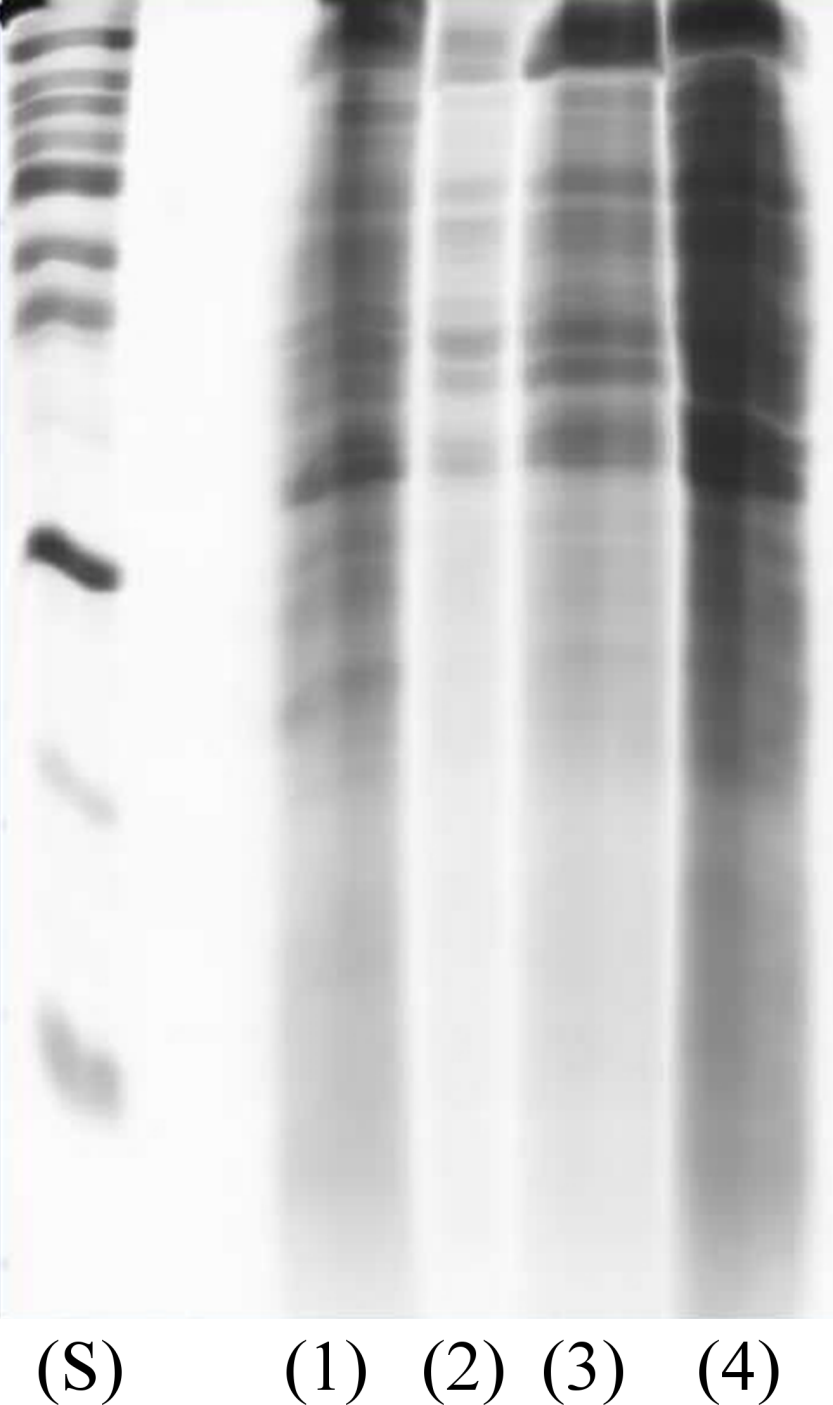
Electrophoretic protein patterns of standard markers (S), *D*. *portulaceae* (1), *Vermisporium-like* (2), *D*. *boerhaaviae (*3) and *E*. *nidulans* (4) extracts, respectively, by SDS-PAGE method with 12% separating gel.

The proteomic analysis of fungal enzymes exhibits several molecules mostly ranging in size from 220 to 20 kDa with clear differences in protein profile’s yield of the A, B, C and D samples as seen in Fig 1.

The fungal enzymes A, B, C and D are good producers of hydrolases. It was found at least two main groups of enzymes produced by these fungi, since each media contains different length carbon chains that lead to different promoter sites of reaction.

The EI values for all fungal crude extracts showed greater potential for the hydrolysis of ceramides (S1 and S2 Figs), leading to the microbial activities. The presence of the enzymes that react with “T80” and “K” media was evidently superior to other produced enzymes, and they are great suppliers of C_18_ chains (Table 1)

**Table 1:** The potential lipolytic activity of extracts expressed as enzymatic index.

	Enzymatic Index			
Fungi	T20	T80	K	W
*A*	3.23^c^ ± 0.18	12.36^b^ ± 4.45	13.04^a^ ± 3.15	1.40^c^ ± 0.13
*B*	2.58^b^ ± 0.40	18.46^a^ ± 3.94	5.10^b^ ± 0.18	1.63^b^ ± 0.04
*C*	3.11^c^ ± 0.86	13.92^a^ ± 2.01	10.92^b^ ± 4.24	4.34^c^ ± 0.52
*D*	2.99^c^ ± 0.18	22.73^a^ ± 0.40	5.45^b^ ± 1.71	2.35^c^ ± 0.72

Similar lower case letters indicate significant similarities between rows, (p < 0.001, ANOVA followed by Tukey test). Fungal enzyme extracts of *Vermisporium-like* corresponds to “A”, of *E*. *nidulans* to “B”, of *D*. *portulaceae* to “C” and of *D*. *boerhaaviae* to “D”. T20, T80, K and W are inductor of enzyme activity in experimental medium.

The ceramides, a lipid group composed by oleic acid (C_18_ chain), is part of the plasma membrane, which plays a fundamental role for cell survival. In the parasite membrane, including *Leishmania* spp, these lipids participate in maintenance and virulence. Therefore, changes in the structure or function of these ceramides in *Leishmania* spp seem to be a promising target in the attempt to eliminate it.

It is known that the leishmanial infection is directly dependent on the lipases action by the membrane’s maintenance mechanism with the rafts synthesis and disruption. The production of extracellular phospholipases increases the virulence of *Leishmania* by stimulation of phosphatidylcholine membrane rafts disruption [19]. leading to the differentiation between of amastigote and promastigote forms.

**Fig 2:**
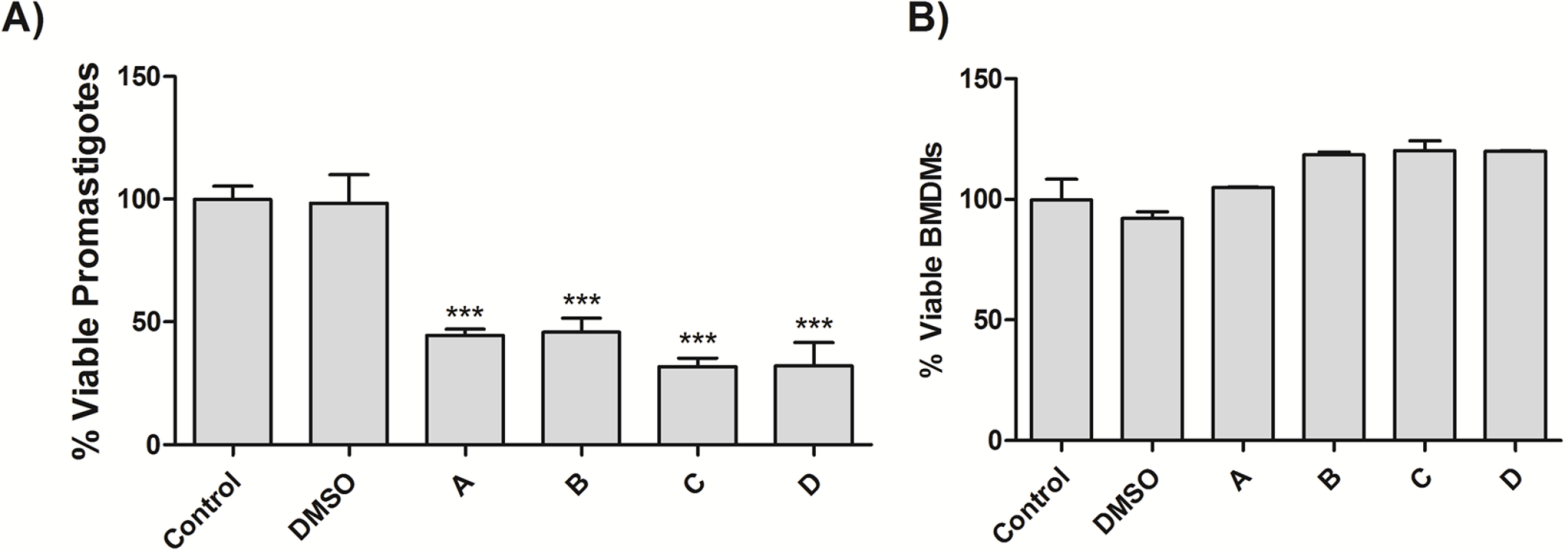
Effect of fungal extracts on *L*. *amazonensis* promastigote growth and BMDMs viability. (A) Growth kinetics of *L*. *amazonensis* promastigote forms (1 x 10^6^) after treatment with fungal extracts (5mg.mL^-1^) or vehicle for 24h. (B) BMDMs (5 x 10^5^/well) were plated in 24-well plates, treated with fungal extract (5mg.mL^-1^) or vehicle and incubated for 24h at 37ºC and 5% CO_2_. DMEM F-12 medium was used as positive control. Data represent the mean ± standard deviation of three independent experiments. *** Significantly different from control (p < 0.0001, ANOVA followed by Tukey test). Fungal enzyme extracts of *Vermisporium-like* corresponds to “A”, of *E*. *nidulans* to “B”, of *D*. *portulaceae* to “C” and of *D*. *boerhaaviae* to “D”.

In the first set of experiments, the antileishmanial effect of fungi crude extracts was investigated against the promastigote forms of *L*. *amazonensis*. All the extracts prevented cell proliferation using 5 mg/mL for 24 h (p<0.0001) (Fig 2), showing the equal antiproliferative effect of standard Glucantime (250 μg/mL) after 24h. After 96 and 168 h, all the extracts in all concentrations reduced cell proliferation (p<0.0001).

Parasites of the genus *Leishmania* are initially recognized by phagocytic cells, and macrophages thereby play an important role in controlling infection and eliminating these intracellular microorganisms. The control of the disease is dependent on the immune response triggered in the host as well as the microbicidal activity of the drug used in the chemotherapy. Despite that activated macrophages have potent microbicidal mechanisms, parasites of the genus *Leishmania* show several virulence factors and are capable of escaping immune responses creating a favorable conditions for proliferation.

In addition, the differentiation of promastigote forms to amastigote is through the transformation of the parasite since it is highly adapted to survive in phagolysosomes, that are rich in triglyceride phospholipids and other lipolytic bioproducts [20]. This mechanism leads the lipase production and the course of the infection in macrophages or neutrophils, allowing the entrance of pathogen toward the defense cells.

**Fig 3:**
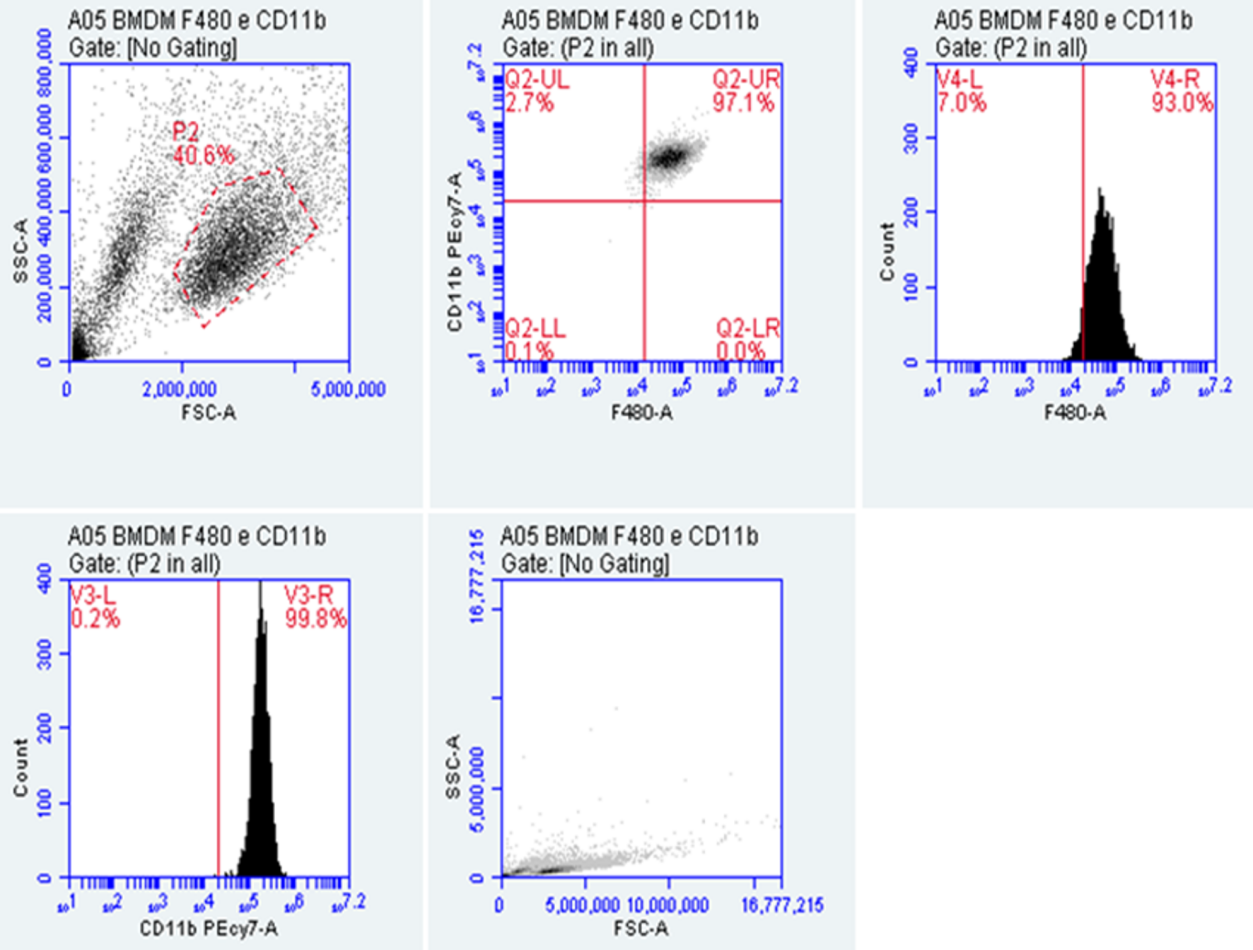
Flow cytometry characterization of bone marrow-derived macrophages (BMDM). Cells were isolated from BALB/c bone marrow and cultured as described (n  =  5 mice). Upon reaching optimal differentiation, macrophages were stained with CD11b-PECy7 and F4/80-PE and analyzed by flow cytometry. Results are representative of three independent experiments.

**Fig 4:**
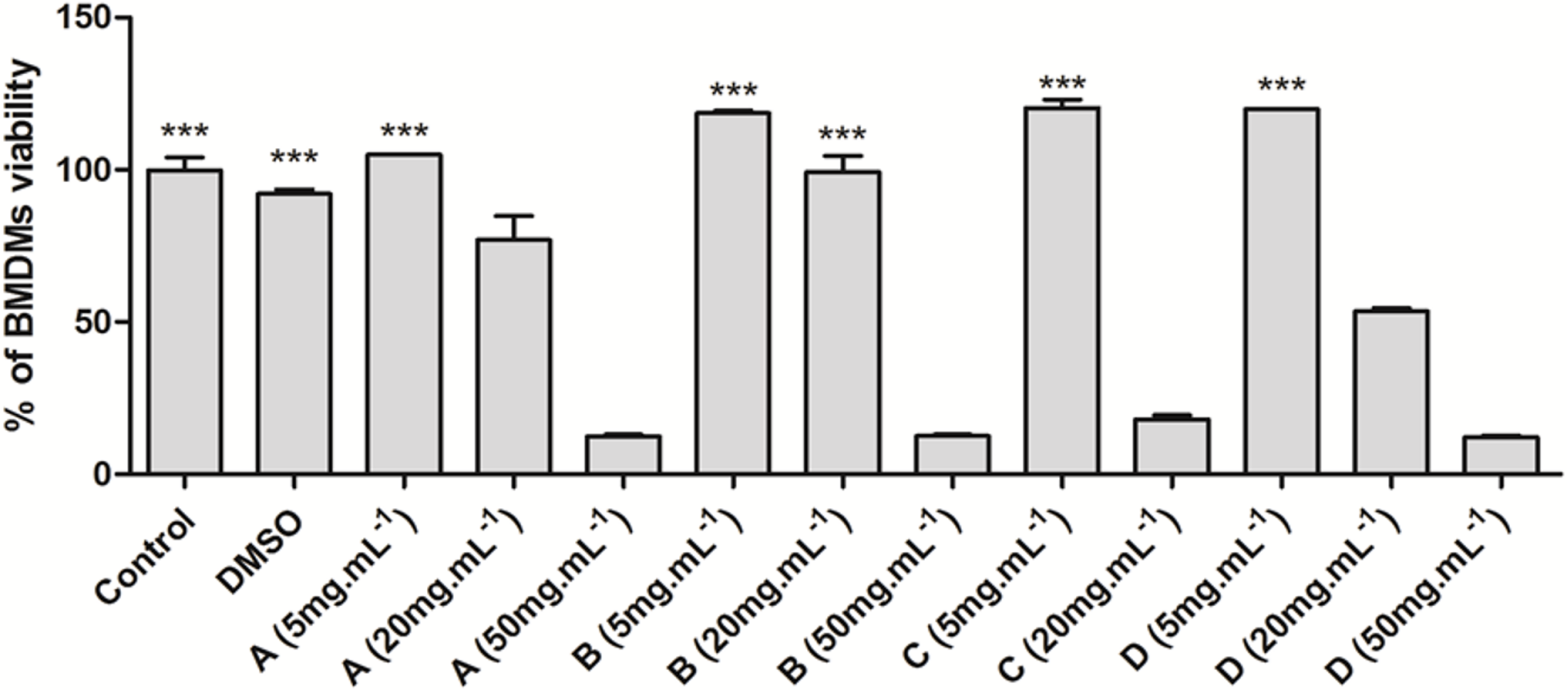
Effect of fungal extracts on BMDMs viability. BMDMs (5 x 10^5^ per well) were plated in 24-well plates, treated with fungal extract (50, 20 and 5 mg.mL^-1^) or vehicle and incubated for 24h at 37ºC and 5% CO_2_. DMEM F-12 medium was used as positive control. Data represent the mean ± standard deviation of three independent experiments. *** Significantly equal to control (p < 0.0001, ANOVA according to Tukey test). Fungal enzyme extracts of *Vermisporium-like* corresponds to “A”, of *E*. *nidulans* to “B”, of *D*. *portulaceae* to “C” and of *D*. *boerhaaviae* to “D”.

Searching for new intervention protocols, tests were performed to verify the integrity of the macrophages cells (Fig 3), and after detecting that these cells reached optimal differentiation to macrophages, all crude extracts were tested at concentrations of 50, 20, and 5mg.mL^-1^ (Fig 4). All fungi crude extracts at a concentration of 5 mg.mL^-1^ do not reduce the macrophage viability (Figs 2 and 4). Only the crude extract “C” does not show a significant difference from the control at a concentration of 20 mg.mL^-1^.

The antileishmanial IC_50_ values for fungal enzymes “A”, “B”, “C” and “D” were respectively of 1.389, 1.120, 1.151 and 0.834 mg.mL^-1^. If they were compared to some plant extracts, all fungi extracts demonstrate medium to low activity, as example *Musa paradisiaca* and *Spondias mombin* whose IC_50_ values were 1.70 and 915 μg.mL^-1^ respectively [21].

**Fig 5:**
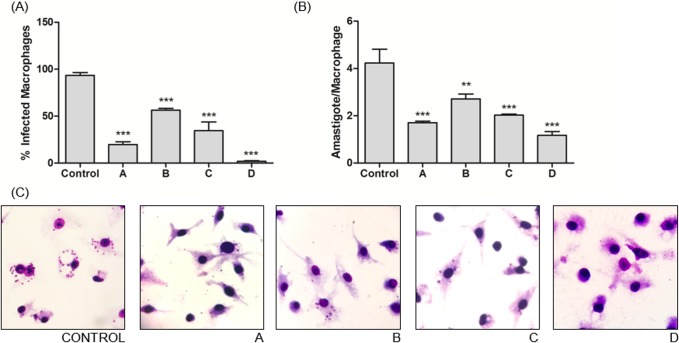
Effect of fungal extracts on *L*. *amazonensis-*infected BMDMs. BMDMs were infected with *L*. *amazonensis* and treated with fungal extracts (5mg.mL^-1^) or vehicle for 24h at 37ºC and 5%CO_2_. (A) Percentage of infected macrophages; (B) a mean number of amastigotes per macrophage. (C) Representative frame images from the effect of each fungal extract on *L*. *amazonensis*-infected BMDMs, where DMEM F-12 was used as a control, *Vermisporium-like* crude extract as “A”, *E*. *nidulans* crude extract as “B”, *D*. *portulaceae* crude extract as “C” and *D*. *boerhaaviae* crude extract as “D”. Data represent the mean ± standard deviation of three independent experiments. **p<0.001, ***p <0.0001 Significantly different from control, according to Tukey test.

The lower concentration (5 mg.mL^-1^) was chosen for testing the 24 h treatment of *Leishmania* infected macrophages, in order to obtain the Selectivity Index (SI). The results of the treatment of *L*. *amazonensis*-infected macrophages with the fungal extracts (A, B, C and D), at a concentration of 5.0 mg.mL^-1^ for 24 h is shown in Fig 5.

The enzymes A, B, C and D were capable of acting directly in the course of infection of *L*. *amazonensis* causing decreases of 78.88, 39.65, 63.17 and 98.13% of infected macrophages, respectively. All the extracts were significantly different from control with p<0.0001. Besides, not only the number or macrophages was reduced but also the intensity of the infection in macrophages.

Moreover, all results of cytotoxic and antileishmanial effects of fungal extracts were confronted to each other, by the Selectivity Index score (Table 2). The IC_50_ values against promastigotes of *L*. *amazonensis* and on macrophages were evaluated. “D” crude extract showed the lower and better IC_50_ values and 20.985 for SI.

**Table 2:** Leishmanicidal (IC_50_ mg.mL^-1^) and cytotoxic effects of fungal extracts.

Fungal Extracts	*L*. *amazonensis* promastigotes	*BMDM*	SI
A	1.388^a^ ± 0.0005	27.175^b^ ± 0.005	19.571^c^
B	1.120^a^± 0.0005	34.360^a^ ± 0.005	30.665^a^
C	0.834^a^± 0.2436	18.580^d^ ± 1.07	23.554^b^
D	1.151^a^± 0.001	22.065^c^ ± 1.235	19.187^c^
**Pentamidine [22]**	23.71^c^	17.90^e^	0.75^d^
	(18.44–30.50)	(0.002–0.026)	
**Glucantime [23]**	13.95^b^ ± 2.06	-	-

BMDM: Bone marrow-derived macrophages, Selectivity Index (SI) = BMDM IC_50_ / promastigote IC_50_

Similar lower case letters indicate significant similarities between columns (p < 0.001, ANOVA followed by Tukey test). Fungal enzyme extracts of *Vermisporium-like* corresponds to “A”, of *E*. *nidulans* to “B”, of *D*. *portulaceae* to “C” and of *D*. *boerhaaviae* to “D”

The fungus antileishmanial results are superior to many isolated and/or modified compounds [24,25], and a good SI of 30.679 was demonstrated by the “B” crude extract. The “A” and “C” crude extracts can also be considered as presenting good leishmanicidal activities with SI of 19.561 and 18.097, respectively.

The antifungal results observed in this study demonstrated that the fungus crude extracts behave similarly with respect to their inhibitory activities (Table 3) at a concentration of 4.88 μg.ml^-1^. Regarding the fungicidal activities, it was observed that extracts from *Dichotomophtora* species showed the better results against skin diseases of animal pathogens. The “C” crude extract was effective against *Malassezia* sp. and “D” crude extract was effective against *M*. *canis* at a concentration of 14.65 μg.mL^-1^.

**Table 3:** Fungicidal activities of fungi extracts.

Extracts	Toxicological activities (μg.mL^-1^)					
	*Malassezia* sp.			*M*. *canis*		
	MIC / MFC			MIC / MFC		
A	4.88^aA^ ± 0.06	/	78.25^cB^ ± 0.88	4.88^aA^ ± 0.06	/	19.53^bB^ ± 1.08
B	4.88^aA^ ± 0.13	/	156.25^cC^ ± 0.18	4.88^aA^ ± 0.01	/	19.53^bB^ ± 1.79
C	4.88^aA^ ± 0.06	/	14.65^bA^ ± 6.91	4.88^aA^ ± 0.06	/	43.94^cC^ ± 3.45
D	4.88^aA^ ± 0.01	/	644.53^cD^ ± 5.96	4.88^aA^ ± 0.03	/	14.65^bA^ ± 6.91

Similar lower case letters indicate significant similarities between rows and upper case letters indicate significant similarities between columns (p < 0.001, ANOVA followed by Tukey test). Fungal enzyme extracts of *Vermisporium-like* corresponds to “A”, of *E*. *nidulans* to “B”, of *D*. *portulaceae* to “C” and of *D*. *boerhaaviae* to “D”

Holetz et al. [26] reported that IC_50_ values below 100 μg.mL^-1^ are indicative of a good antimicrobial activity and above 500 weak activity, and between these values the activity is considered moderate. Weak activity denotes not relevant for use in pharmaceutical treating of fungal infections. Using these IC_50_ parameters the extract “A” had a good antifungal action against *Malassezia* sp. with a medium concentration of 78.25 μg.ml^-1^. The extract “B” has a moderate activity at a concentration of 156.25 μg.ml^-1^. The “D” extract demonstrated a weak activity with IC_50_ equal 644.53 μg.ml^-1^.

With regard to fungicidal capacity against *M*. *canis*, all the extracts demonstrated good activity mainly “A” and “B” extracts.

As the most dermatophytes demonstrate lipid-addict, during an infection there is a great expression of pathogen’s lipases [27]. Besides the mechanism of action of several compounds during the disease caused by a dermal pathogen remains unknown [6] the lipases are effective targets, as observed in this work. The use of external lipases, produced by another microorganism, can be a way to disrupt the course of infection, by causing a gradual decline of cell membrane pathogen homeostasis.

## Conclusions

This work evidences the presence of lipases in crude extracts obtained from culture with the endophytic fungi: *Vermisporium*-like, *Emericella nidullans*, *Dichotomophtora boerhaaviae* and *Dichotomophtora portulaceae*, with clear differences in protein profiles. With respect to leishmanicidal activity, it is possible to infer that lipases from the enzymatic extracts of *Dichotomophtora portulacae* and *E*. *nidullans*, are more effective on promastigote forms of *L*. *amazonensis*. With respect to fungicidal activity, it was found that the *Dichotomophtora* species produced the most effective enzymes against the studied fungi followed by the *Vermisporium*-like species, which presented the lowest MIC / MFC for the dermatophytes studied.

Although the mechanism of action of these enzymes was not completely elucidated, evidences pointing to some effect on membrane lipids in all pathogens. Nevertheless, further investigation is necessary to understand how these protein products potentially act in pathogen’s enzymes through *de novo* synthesis and membrane modifications. These modifications are seen as a virulence factor, which plays an important role at cell metabolism maturation in the host.

## Supporting information

S1 Fig Enzymatic activity plate assay experimental concept. Determination of Hydrolases.
(TIF)

S2 Fig Determination of lipolytic activity of extracts plate assay experimental concept. Determination of Hydrolases.
(TIF)

S1 Table The potential lipolytic activity at plate assay expressed as enzymatic index. (XLSX)

S2 Table Leishmanicidal (IC50 mg.mL-1) and cytotoxic effects and selectivity index of fungal extracts. GraphPad Prism 7 statistical files compressed as ZIP file archieve.
(RAR)
