# Supplementary figures and images for: Leishmanicidal and fungicidal activity of lipases obtained from endophytic fungi extracts
Source: PLoS One. 2018 Jun 18;13(6):e0196796. doi: 10.1371/journal.pone.0196796 (PMC6005525; doi:10.1371/journal.pone.0196796)

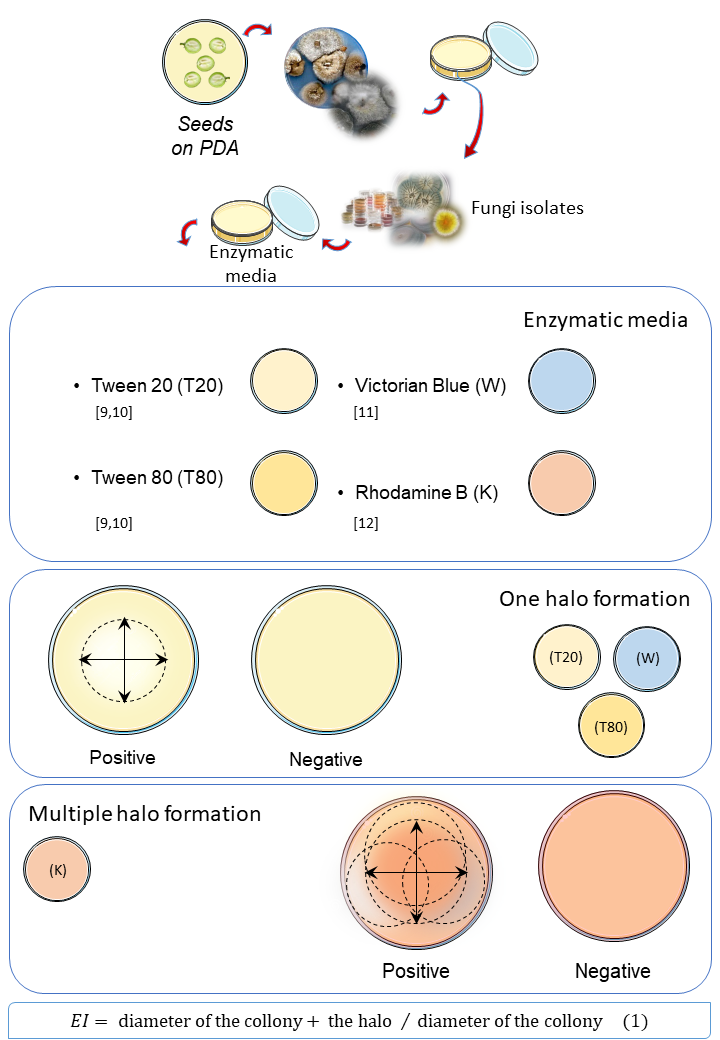

Supplement: S1 Fig — Determination of Hydrolases. (TIF) [file pone.0196796.s001.tif]

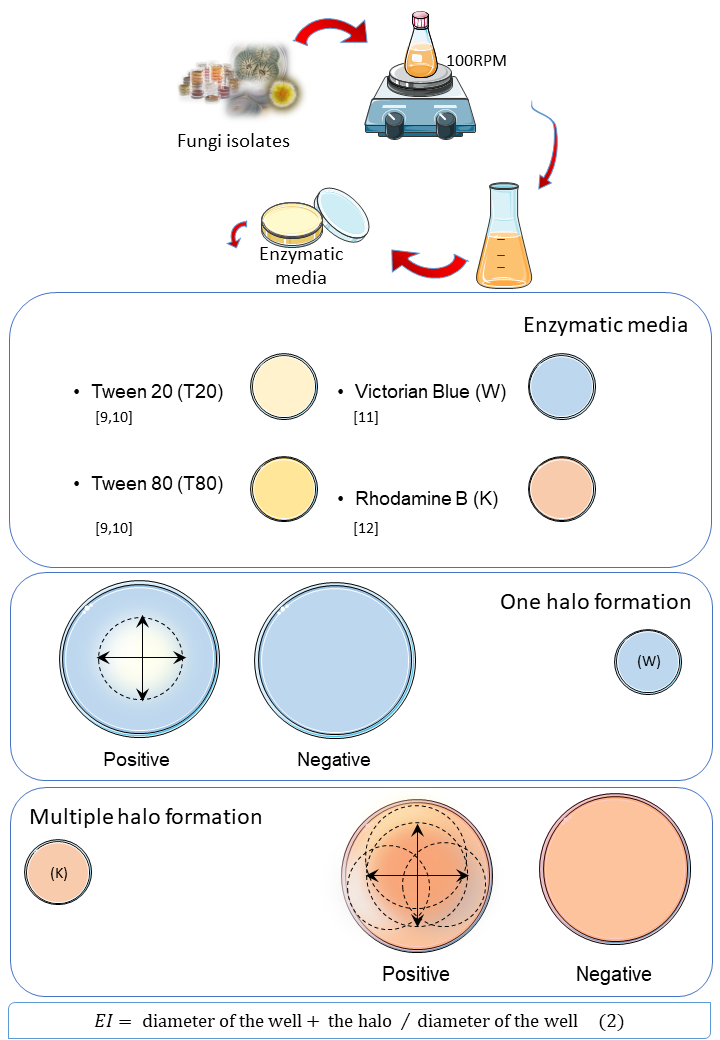

Supplement: S2 Fig — Determination of Hydrolases. (TIF) [file pone.0196796.s002.tif]
